# Confounding effects of blood hemoglobin and hematocrit levels on flap perfusion measurement with the Oxygen-to-see (O2C) analysis system in microvascular head and neck reconstruction– a retrospective study

**DOI:** 10.1186/s12893-025-02888-8

**Published:** 2025-04-08

**Authors:** Mark Ooms, Philipp Winnand, Marius Heitzer, Anna Bock, Marie Katz, Johannes Bickenbach, Frank Hölzle, Ali Modabber

**Affiliations:** 1 Department of Oral and Maxillofacial Surgery, University Hospital RWTH Aachen, Pauwelsstraße 30, 52074 Aachen, Germany; 2 Department of Intensive Care Medicine, Uniklinik RWTH Aachen, Pauwelsstraße 30, 52074 Aachen, Germany

**Keywords:** Microvascular head and neck reconstruction, Free flap, Flap monitoring, Flap perfusion measurement, Oxygen-to-see (O2C) analysis system, Blood hemoglobin, Blood hematocrit, Blood flow, Hemoglobin concentration, Hemoglobin oxygen saturation.

## Abstract

**Background:**

The Oxygen-2-see (O2C) analysis system can measure flap perfusion, which is a prerequisite for flap viability, and it is therefore commonly used in flap monitoring for microvascular head and neck reconstruction. However, in the context of predefined threshold values for perfusion parameters indicating vascular flap compromise, it is unclear whether blood hemoglobin and hematocrit levels are confounding variables. The aim of this study was to investigate the influence of blood hemoglobin and hematocrit levels on flap perfusion parameters.

**Methods:**

Perfusion parameters (i.e., flap blood flow, hemoglobin concentration, and hemoglobin oxygen saturation) measured intraoperatively and postoperatively with the O2C analysis system at 8- and 2-mm tissue depths were retrospectively analyzed in 125 patients reconstructed with a radial free forearm flap (RFFF), an anterolateral thigh flap (ALTF), or a fibula free flap (FFF) between 2011 and 2020. Associations between perfusion parameters and blood hemoglobin and hematocrit levels were analyzed using Spearman correlation coefficient and multiple linear regression models.

**Results:**

Postoperative hemoglobin concentration at a 2-mm tissue depth was associated with blood hemoglobin and hematocrit levels in RFFFs (*r* = 0.259, *p* = 0.031; and *r* = 0.268, *p* = 0.026; respectively). Both associations persisted in multivariable regression analysis (*p* = 0.040 and *p* = 0.036). No other associations between perfusion parameters and blood hemoglobin and hematocrit levels were observed for RFFFs, ALTFs or FFFs (all *p* > 0.05).

**Conclusions:**

For the perfusion parameters flap blood flow and hemoglobin oxygen saturation no association with blood hemoglobin or hematocrit levels was observed. This underlines the validity of absolute threshold levels for indicating vascular flap compromise in the context of flap monitoring with the O2C analysis system.

## Background

Reconstruction of complex defects in the head and neck region is routinely performed with microvascular free flaps, resulting in excellent outcomes and high success rates [1–3]. However, flap failure occurs, which is why postoperative flap monitoring is considered essential for the timely detection and correction of flap failure due to vascular flap compromise in microvascular reconstruction [1, 3–5].

The Oxygen-2-see (O2C) analysis system (LEA Medizintechnik) can be used for the purpose of flap monitoring based on the measurement of flap perfusion at 8- and 2-mm tissue depths with respect to flap blood flow, hemoglobin concentration, and hemoglobin oxygen saturation, and it is capable of the timely detection of flap failure based on threshold values indicating vascular flap compromise [4, 6, 7]. These threshold values are absolute for flap blood flow and hemoglobin oxygen saturation and relative for hemoglobin concentration [6, 7]. However, it is unclear whether blood hemoglobin and hematocrit levels have an influence on flap perfusion parameters, which may call the validity of the predefined threshold values in question, particularly absolute threshold values for flap blood flow and hemoglobin oxygen saturation, which are used for clinical decision-making on flap revision [6, 7].

Blood hemoglobin and hematocrit levels show high variability in patients during microvascular reconstructions in the head and neck region due to the loss and substitution of blood and fluids [8–10]. Furthermore, the influence of blood hemoglobin and hematocrit levels on flap perfusion parameters is conceivable because higher blood hemoglobin and hematocrit levels are associated with increased blood viscosity and flow resistance and increased blood oxygen-carrying capacity, respectively, which may lead to lower blood flow and higher hemoglobin oxygen saturation [11–16]. Indeed, an animal study showed that lower blood hematocrit levels improved microvascular free flap perfusion [17].

This study aimed to investigate the relationship between blood hemoglobin and hematocrit levels and flap perfusion parameters as measured with the O2C analysis system in microvascular head and neck reconstruction.

## Methods

### Study population

This retrospective study, which was based on a dataset collected in the past for routine clinical purposes, was approved by the local ethics committee of the Medical Faculty RWTH Aachen University (EK 309 − 20).

The study population consisted of 125 patients reconstructed with a radial free forearm flap (RFFF), an anterolateral thigh flap (ALTF), or a fibula free flap (FFF) in the head and neck region after malignant or nonmalignant diseases in our Department of Oral and Maxillofacial Surgery between 2011 and 2020. The exclusion criteria for patients were incomplete data records, age under 18 years, and blood transfusion or the substitution of colloidal volume between the blood gas analysis and the measurement of flap perfusion.

Comorbidities were defined based on discipline-specific guidelines, and smoking was defined as actual or past daily smoking for a period of at least six months [18]. The status of prior neck dissection or irradiation was defined as positive if the patient had undergone anatomic dissection of the recipient vessel corresponding to a neck dissection or irradiation to the recipient vessel area corresponding to a neck irradiation, respectively. The durations of surgery and flap ischemia were defined as the time between the first incision and the last suture and between the interruption of flap perfusion at the donor site and the onset of flap perfusion at the recipient site. The status of flap revision was defined as positive if the patient had surgical revision of the anastomosis, with a return to the operating room, and the status of flap success was defined as negative if the patient had the flap removed because of flap necrosis.

The surgical procedures were conducted under general anesthesia in the operation room. The arterial and venous anastomoses were performed in an end-to-end fashion and in end-to-side or end-to-end fashion, respectively. Postoperatively, all patients remained in the intensive care unit at least until the next morning, with invasive mechanical ventilation, analgosedation, invasive arterial blood pressure monitoring, and blood pressure regulation via central venous norepinephrine administration as needed (target systolic pressure above 125 mmHg).

### Blood laboratory parameter data

Intraoperative and postoperative blood hemoglobin and hematocrit levels were determined via arterial blood gas analysis.

### Flap perfusion measurement data

Flap perfusion was measured with the O2C tissue oxygen analysis system (Oxygen-2-see, LEA Medizintechnik, Giesen, Germany) for 10 s (with a lead time of 4 s), with ambient light compensation control at 8- and 2-mm tissue depths intraoperatively (after the release of the anastomosis in the operating room) and postoperatively (on the first postoperative morning in the intensive care unit), with the probe being placed centrally on the dried skin portion of the flap in a sterile sheath. The perfusion parameters were determined as follows: blood flow (arbitrary units [AU]) was determined by analyzing the Doppler shift of the laser light due to the erythrocyte movement as the product of erythrocyte quantity and erythrocyte velocity based on laser Doppler spectroscopy (830 nm; 30 mW), hemoglobin concentration (AU) was determined by analyzing the sum of the absorbances of the white light based on white light spectroscopy (500–800 nm; 50 W), and hemoglobin oxygen saturation (%) was determined by analyzing the color change of the white light in comparison to reference hemoglobin spectra with known oxygen saturation based on white light spectroscopy (500–800 nm; 50 W) [6, 19].

### Statistical analysis

Patients were divided into groups based on flap type (RFFF, ALTF, or FFF). Associations between blood hemoglobin or hematocrit levels and flap perfusion parameters were analyzed separately for each flap type using Spearman correlation coefficient. In the case of significant results, these were further analyzed, after a stepwise multiple regression analysis to identify significant predictors among baseline data (included: ischemia duration *p* = 0.008; excluded: sex *p* = 0.154, age *p* = 0.063, BMI *p* = 0.216, ASA *p* = 0.604, flap location *p* = 0.067, arterial anastomosis recipient vessel *p* = 0.128, surgery duration *p* = 0.617, diabetes *p* = 0.116, arterial hypertension *p* = 0.397, smoking status *p* = 0.796, prior neck dissection *p* = 0.792, prior neck irradiation *p* = 0.603, flap revision *p* = 0.193) using multiple regression analysis adjusted for ischemia duration (min), mean arterial blood pressure (mmHg), and administered catecholamine dose (µg/min per kg). Levels of *p* < 0.05 were considered statistically significant without performing adjustment for multiple testing. The statistical analysis was performed using SPSS Version 28 (SPSS, IBM, New York, USA).

## Results

### Clinical characteristics of the study population

The study population consisted of 125 patients (69 patients reconstructed with RFFFs, 31 with ALTFs, and 25 with FFFs) (Table 1). Three RFFFs, one ALTF, and four FFFs were revised due to venous congestion.

**Table 1:** Clinical characteristics of the study population

Variable	RFFF (*n* = 69)	ALTF (*n* = 31)	FFF (*n* = 25)
***Sex*** *(n)*			
male	46 (66.7%)	16 (51.6%)	8 (32.0%)
female	23 (33.3%)	15 (48.4%)	17 (68.0%)
***Age*** *(years)*	65.0 (15.0)	62.0 (22.0)	56.0 (16.0)
***BMI*** *(kg/m²)*	26.7 (6.8)	24.2 (8.2)	23.6 (5.2)
***ASA*** *(n)*			
1 + 2	42 (60.9%)	17 (54.8%)	13 (52.0%)
3 + 4	27 (39.1%)	14 (45.2%)	12 (48.0%)
***Flap location*** *(n)*			
tongue	15 (21.7%)	2 (6.5%)	0 (0.0%)
floor of mouth	18 (26.1%)	4 (12.9%)	0 (0.0%)
mandible	5 (7.2%)	12 (38.7%)	21 (84.0%)
maxilla + hard palate	10 (14.5%)	5 (16.1%)	3 (12.0%)
cheek	7 (10.1%)	1 (3.2%)	0 (0.0%)
soft palate	4 (5.8%)	2 (6.5%)	0 (0.0%)
extraoral	10 (14.5%)	5 (16.1%)	1 (4.0%)
***Arterial anastomosis recipient vessel*** *(n)*			
ECA	4 (5.8%)	1 (3.2%)	4 (16.0%)
FAA	27 (39.1%)	12 (38.7%)	10 (40.0%)
LIA	2 (2.9%)	2 (6.5%)	3 (12.0%)
STA	36 (52.2%)	16 (51.6%)	8 (32.0%)
***Surgery duration*** *(min)*	513.0 (150.0)	528.0 (91.0)	545.0 (93.0)
***Flap ischemia duration*** *(min)*	108.0 (28.0)	105.0 (37.0)	120.0 (38.0)
***Diabetes*** *(n)*			
no	60 (87.0%)	21 (67.7%)	22 (88.0%)
yes	9 (13.0%)	10 (32.3%)	3 (12.0%)
***Arterial hypertension*** *(n)*			
no	42 (60.9%)	18 (58.1%)	18 (72.0%)
yes	27 (39.1%)	13 (41.9%)	7 (28.0%)
**Smoking status** *(n)*			
no	42 (60.9%)	17 (54.8%)	15 (60.0%)
yes	27 (39.1%)	14 (45.2%)	10 (40.0%)
**Prior neck dissection** *(n)*			
no	61 (88.4%)	23 (74.2%)	12 (48.0%)
yes	8 (11.6%)	8 (25.8%)	13 (52.0%)
**Prior neck irradiation** *(n)*			
no	66 (95.7%)	28 (90.3%)	19 (76.0%)
yes	3 (4.3%)	3 (9.7%)	6 (24.0%)
**Flap survival** *(n)*			
no	0 (0.0%)	0 (0.0%)	2 (8.0%)
yes	69 (100.0%)	31 (100.0%)	23 (92.0%)
**Flap revision** *(n)*			
no	66 (95.7%)	30 (96.8%)	21 (84.0%)
yes	3 (4.3%)	1 (3.2%)	4 (16.0%)

Parameters are indicated as numbers (with percentage) for categorical data (sex, ASA, flap location, arterial anastomosis recipient vessel, diabetes, arterial hypertension, smoking status, prior neck dissection, prior neck irradiation, flap survival, flap revision) or median (with interquartile range) for metric data (age, BMI, surgery duration, flap ischemia duration) (separately described for patients reconstructed with a RFFF, ALTF or FFF); abbreviations: RFFF = radial free forearm flap, ALTF = anterolateral thigh flap, FFF = fibula free flap, BMI = body mass index, ASA = American Society of Anesthesiologists score, ECA = external carotid artery, FAA = facial artery, LIA = lingual artery, STA = superior thyroid artery

### Blood hemoglobin and hematocrit levels

The median blood hemoglobin and hematocrit levels related to intraoperative flap perfusion measurement in patients reconstructed with RFFFs, ALTFs, or FFFs were as follows: 10.3 g/dL, 9.5 g/dL, and 9.5 g/dL and 32.0%, 29.8%, and 29.7%, respectively (Table 2). The median blood hemoglobin and hematocrit levels related to postoperative flap perfusion measurement in patients reconstructed with RFFFs, ALTFs, or FFFs were as follows: 9.8 g/dL, 9.2 g/dL, and 8.7 g/dL and 30.0%, 28.7%, and 26.7%, respectively.

**Table 2:** Blood hemoglobin and hematocrit levels

Variable	RFFF (*n* = 69)	ALTF (*n* = 31)	FFF (*n* = 25)
**Intraoperative measurement**			
**Hemoglobin** (g/dL)	10.3 (2.1)	9.5 (2.0)	9.5 (1.5)
**Hematocrit** (%)	32.0 (6.6)	29.8 (5.8)	29.7 (4.1)
**Postoperative measurement**			
**Hemoglobin** (g/dL)	9.8 (1.7)	9.2 (1.7)	8.7 (0.9)
**Hematocrit** (%)	30.0 (5.3)	28.7 (4.7)	26.7 (2.6)

Parameters are indicated as median (with interquartile range) for hemoglobin concentration (g/ dL) and hematocrit levels (%) according to arterial blood gas analysis and used as reference values for the flap perfusion measurement (separately described for patients reconstructed with a RFFF, ALTF or FFF); abbreviations: RFFF = radial free forearm flap, ALTF = anterolateral thigh flap, FFF = fibula free flap

The median time interval between blood gas analysis and flap perfusion measurement was 32 min intraoperatively and 53 min postoperatively. The median volume substitution during this time interval was 2.9 ml/min intraoperatively and 2.0 ml/min postoperatively.

### Association between flap perfusion parameters and blood hemoglobin levels

**Fig. 1:**
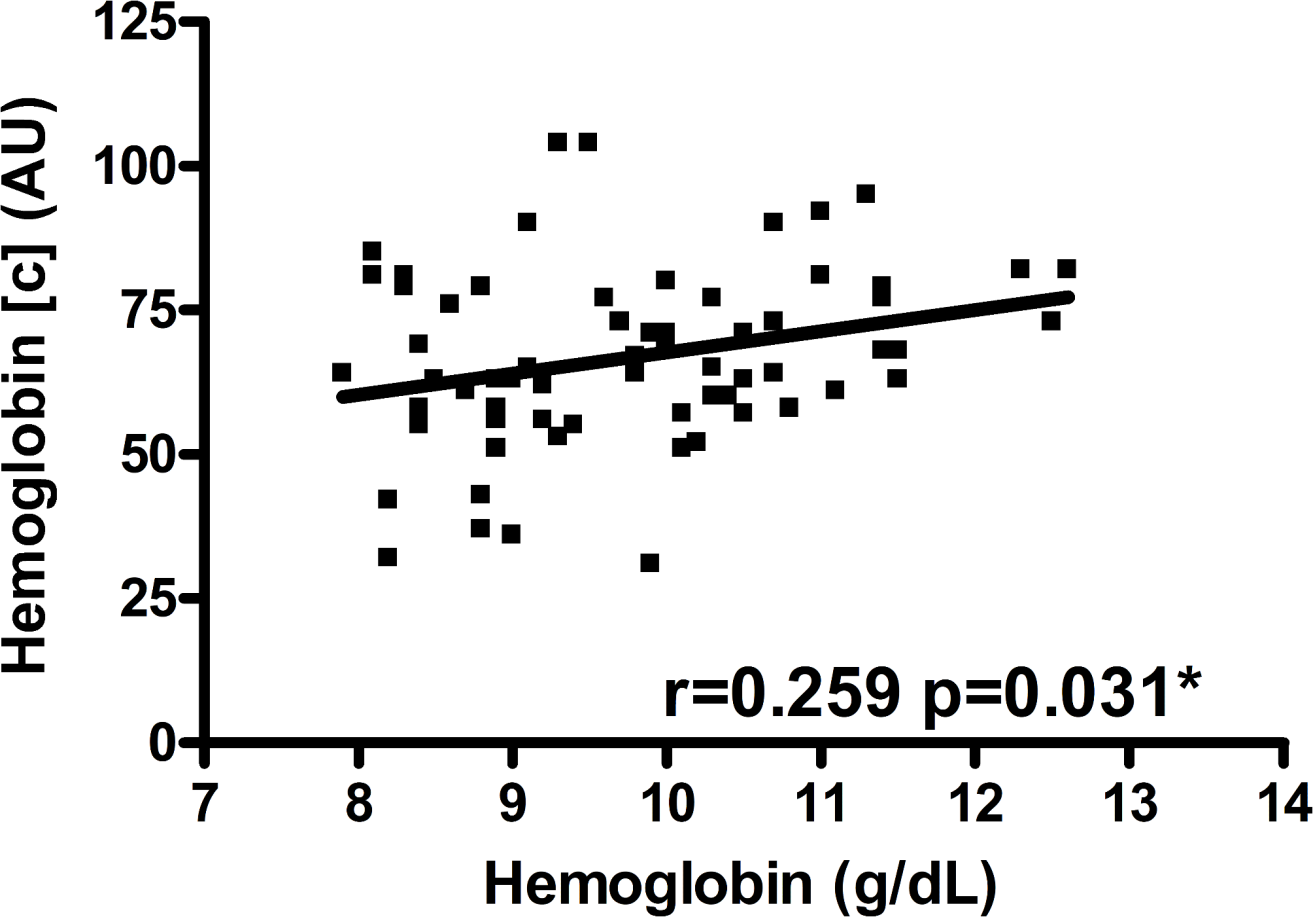
Postoperative hemoglobin concentration and blood hemoglobin levels in RFFFs Scatter plot for postoperative hemoglobin concentration (AU) at 2-mm tissue depth and blood hemoglobin level in RFFFs; r and p-value corresponding to Spearman correlation coefficient (*p = 0.040 upon adjustment for ischemia duration (min), mean arterial blood pressure (mmHg) and administered catecholamine dose (µg/min per kg) in multiple linear regression analysis); abbreviations: [c] = concentration, AU = arbitrary units

Postoperative hemoglobin concentration in a 2-mm tissue depth was positively correlated with blood hemoglobin level in RFFFs (*r* = 0.259, *p* = 0.031) (Table 3; Fig. 1). This association persisted in multivariable testing adjusting for ischemia duration, mean arterial blood pressure and administered catecholamine dose (*p* = 0.040) (Table 4). Postoperative hemoglobin concentration in a 8-mm tissue depth was not correlated with blood hemoglobin level in RFFFs (*r* = 0.103, *p* = 0.400) (Table 3).

**Table 3:** Association between flap perfusion parameters and blood hemoglobin levels

Variable	RFFF (*n* = 69)		ALTF (*n* = 31)		FFF (*n* = 25)	
	r	*p*-value	r	*p*-value	r	*p*-value
**Intraoperative measurement**						
**Flow** (AU)						
**8 mm**	0.228	0.060	0.162	0.384	0.026	0.903
**2 mm**	0.055	0.656	0.157	0.398	-0.118	0.574
**Hemoglobin concentration** (AU)						
**8 mm**	0.172	0.158	-0.142	0.445	-0.215	0.302
**2 mm**	0.212	0.080	0.209	0.259	0.269	0.193
**Hemoglobin oxygen saturation** (%)						
**8 mm**	0.027	0.826	0.137	0.464	-0.013	0.952
**2 mm**	0.101	0.409	0.214	0.247	0.061	0.773
**Postoperative measurement**						
**Flow** (AU)						
**8 mm**	0.128	0.293	-0.006	0.974	0.122	0.562
**2 mm**	-0.014	0.912	-0.229	0.215	-0.010	0.961
**Hemoglobin concentration** (AU)						
**8 mm**	0.103	0.400	-0.147	0.430	0.377	0.063
**2 mm**	**0.259**	0.031*	0.270	0.142	0.179	0.391
**Hemoglobin oxygen saturation** (%)						
**8 mm**	0.040	0.742	-0.153	0.410	-0.123	0.558
**2 mm**	0.084	0.495	0.046	0.804	-0.022	0.915

Parameters are indicated as Spearman correlation coefficient (r) calculated between flap perfusion parameters (flow, hemoglobin concentration, hemoglobin oxygen saturation) at 8- and 2-mm tissue depth and blood hemoglobin levels (separately described for patients reconstructed with a RFFF, ALTF or FFF); p-values corresponding to calculation of Spearman correlation coefficient; significant p-values are bold (*p = 0.040 upon adjustment for ischemia duration (min), mean arterial blood pressure (mmHg) and administered catecholamine dose (µg/min per kg) in multiple regression analysis); abbreviations: RFFF = radial free forearm flap, ALTF = anterolateral thigh flap, FFF = fibula free flap, AU = arbitrary units

**Table 4:** Multiple regression analysis

Variable	B (with CI)	*p*-value
**Postoperative measurement - Hemoglobin concentration (AU) − 2-mm tissue depth**		
**Hemoglobin (g/dL)**	3.223 (0.151 - 6.294)	**0.040**
**Ischemia duration (min)**	-0.183 (-0.334 - -0.032)	**0.019**
**Mean arterial blood pressure (mmHg)**	-0.142 (-0.484 - 0.200)	0.411
**Administered catecholamine dose (µg/min per kg)**	28.529 (-17.628 - 74.686)	0.221
**Postoperative measurement - Hemoglobin concentration (AU) − 2-mm tissue depth**		
**Hematocrit (%)**	1.095 (0.075 - 2.114)	**0.036**
**Ischemia duration (min)**	-0.184 (-0.335 - -0.033)	**0.017**
**Mean arterial blood pressure (mmHg)**	-0.133 (-0.475 - 0.208)	0.439
**Administered catecholamine dose (µg/min per kg)**	28.238 (-17.826 - 74.302)	0.225

Parameters are indicated as B regression coefficients (with 95% confidence interval) and p-values corresponding to multiple regression analysis for postoperative measurement of hemoglobin concentration (AU) in 2-mm tissue depth separately for hemoglobin concentration (g/dL) upon adjustment for ischemia duration (min), mean arterial blood pressure (mmHg), and administered catecholamine dose (µg/min per kg), and for hematocrit levels (%) upon adjustment for ischemia duration (min), mean arterial blood pressure (mmHg), and administered catecholamine dose (µg/min per kg) in patients reconstructed with a RFFF; abbreviations: CI = confidence interval; AU = arbitrary units

No further association was found between flap perfusion parameters and blood hemoglobin levels (all *p* > 0.05).

### Association between flap perfusion parameters and blood hematocrit levels

**Fig. 2:**
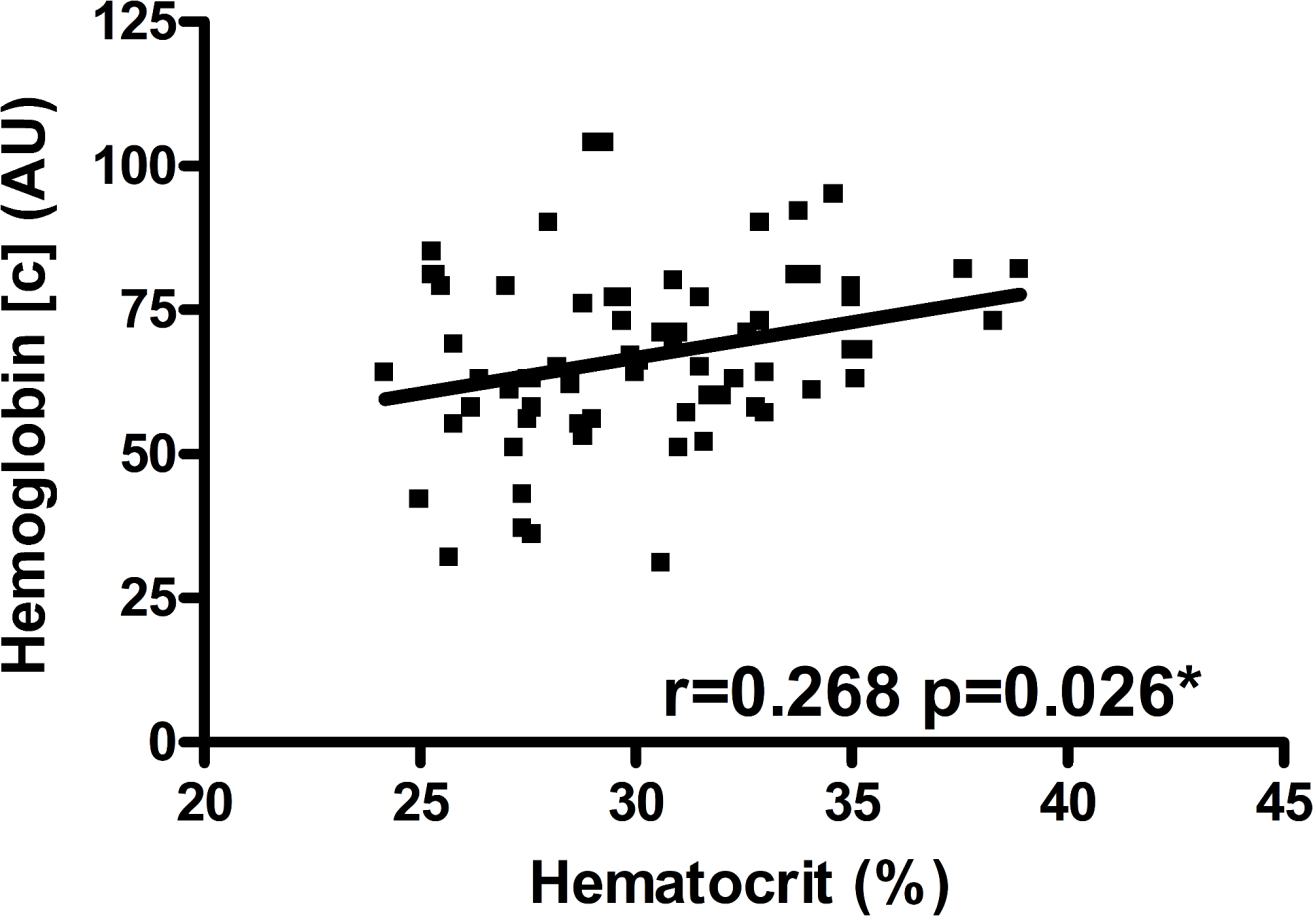
Postoperative hemoglobin concentration and blood hematocrit levels in RFFFs Scatter plot for postoperative hemoglobin concentration (AU) at 2-mm tissue depth and blood hematocrit level in RFFFs; r and p-value corresponding to Spearman correlation coefficient (*p = 0.036 upon adjustment for ischemia duration (min), mean arterial blood pressure (mmHg) and administered catecholamine dose (µg/min per kg) in multiple linear regression analysis); abbreviations: [c] = concentration, AU = arbitrary units

Postoperative hemoglobin concentration in a 2-mm tissue depth was positively correlated with blood hematocrit level in RFFFs (*r* = 0.268, *p* = 0.026) (Table 5; Fig. 2). This association persisted in multivariable testing adjusting for ischemia duration, mean arterial blood pressure and administered catecholamine dose (*p* = 0.036) (Table 4). Postoperative hemoglobin concentration in a 8-mm tissue depth was not correlated with blood hematocrit level in RFFFs (*r* = 0.093, *p* = 0.447) (Table 5).

**Table 5:** Association between flap perfusion parameters and blood hematocrit levels

Variable	RFFF (*n* = 69)		ALTF (*n* = 31)		FFF (*n* = 25)	
	r	*p*-value	r	*p*-value	r	*p*-value
**Intraoperative measurement**						
**Flow** (AU)						
**8 mm**	0.231	0.056	0.140	0.452	0.068	0.748
**2 mm**	0.036	0.772	0.177	0.342	-0.145	0.489
**Hemoglobin concentration** (AU)						
**8 mm**	0.175	0.150	-0.167	0.369	-0.276	0.182
**2 mm**	0.190	0.117	0.311	0.089	0.285	0.168
**Hemoglobin oxygen saturation** (%)						
**8 mm**	0.011	0.931	0.040	0.832	-0.080	0.703
**2 mm**	0.080	0.513	0.304	0.096	-0.033	0.877
**Postoperative measurement**						
**Flow** (AU)						
**8 mm**	0.102	0.405	-0.021	0.909	0.172	0.411
**2 mm**	-0.035	0.776	-0.236	0.202	0.017	0.935
**Hemoglobin concentration** (AU)						
**8 mm**	0.093	0.447	-0.153	0.412	0.340	0.096
**2 mm**	**0.268**	0.026*	0.246	0.183	0.159	0.448
**Hemoglobin oxygen saturation** (%)						
**8 mm**	0.016	0.899	-0.128	0.491	-0.100	0.635
**2 mm**	0.088	0.473	0.072	0.701	0.066	0.754

Parameters are indicated as Spearman correlation coefficient (r) calculated between flap perfusion parameters (flow, hemoglobin concentration, hemoglobin oxygen saturation) at 8- and 2-mm tissue depth and blood hematocrit levels (separately described for patients reconstructed with a RFFF, ALTF or FFF); p-values corresponding to calculation of Spearman correlation coefficient; significant p-values are bold (*p = 0.036 upon adjustment for ischemia duration (min), mean arterial blood pressure (mmHg) and administered catecholamine dose (µg/min per kg) in multiple regression analysis); abbreviations: RFFF = radial free forearm flap, ALTF = anterolateral thigh flap, FFF = fibula free flap, AU = arbitrary units

No further association was found between flap perfusion parameters and blood hematocrit levels (all *p* > 0.05).

## Discussion

This study investigated the relationships between blood hemoglobin and hematocrit levels and flap perfusion, as both of these blood parameter levels vary perioperatively and can influence flap perfusion, and as flap perfusion is used as a parameter in flap monitoring [4, 6–17].

The O2C analysis system is frequently used for flap monitoring in microvascular head and neck reconstruction for the timely detection and subsequent correction of vascular flap compromise to facilitate flap salvage, and it is based on the measurement of flap perfusion parameters (i.e., flap blood flow, hemoglobin concentration, and hemoglobin oxygen saturation) in relation to predefined threshold values indicating vascular flap compromise [1, 4–7]. The threshold values for flap blood flow and hemoglobin oxygen saturation at 8- and 2-mm tissue depths are absolute: for RFFFs < 20/10 AU and < 15%, for ALTFs < 15/5 AU and < 10%, and for FFFs < 15/5 AU and < 10% [6, 7]. In contrast, the threshold values for hemoglobin concentration at 8- and 2-mm tissue depths are relative: for RFFFs > 30%, for ALTFs > 30%, and for FFFs > 30% [6, 7]. However, these threshold values do not take blood hemoglobin and hematocrit levels into account, although both blood parameter levels show high variability perioperatively in the context of microvascular free flap reconstruction because of the loss and subsequent substitution of blood and fluids, and both parameters can potentially influence flap perfusion [8–17]. Blood hemoglobin and hematocrit levels (i.e., the concentration of hemoglobin and the percentage of cellular blood components, especially erythrocytes) may influence flap perfusion and flap perfusion parameters directly in terms of hemoglobin concentration and indirectly in terms of flap blood flow and hemoglobin oxygen saturation, as increases in both blood parameter levels theoretically decrease flap blood flow due to increased blood viscosity and flow resistance and increase hemoglobin concentration and, thus, the hemoglobin oxygen-carrying capacity [11, 15, 16]. Such an influence could affect the validity of the threshold values used for flap monitoring with the O2C analysis system and thus decision-making about flap revision, particularly regarding the absolute threshold values for flap blood flow and hemoglobin oxygen saturation [6, 7]. However, evaluating the validity of the absolute predefined threshold values for various blood hemoglobin and hematocrit levels is impeded by the low flap revision rate [2, 3].

This study showed that only postoperative hemoglobin concentration at a 2-mm tissue depth was associated with blood hemoglobin and hematocrit levels in RFFFs, while other associations were not observed in RFFFs, ALTFs, or FFFs.

The positive associations between the flap perfusion parameter hemoglobin concentration at a 2-mm tissue depth and blood hemoglobin and hematocrit level in RFFFs are comprehensible, as the O2C analysis system measures the hemoglobin in the flap vasculature, the quantity of which is directly reflected by the blood hemoglobin level and indirectly reflected by the hematocrit level, which represents the percentage of cellular blood components, especially hemoglobin-carrying erythrocytes [6, 15]. The vascular anatomy of the RFFF, which is a fasciocutaneous flap with multiple connection vessels and an increasing cross-sectional area from the source vessel to the flap tissue, in contrast to the ALTF and FFF, which are perforator flaps, could contribute to the observed association, as the RFFF acts more like a vascular shunt, reflecting blood hemoglobin and hematocrit levels more directly [20–25]. Furthermore, differences between flap types in postoperative perfusion dynamics observed in previous studies, such as higher blood flow in RFFFs, may also contribute to the observed associations [6, 7]. However, both associations were quantitatively weak [26]. The lack of a relationship between blood hematocrit levels and the perfusion parameters blood flow and hemoglobin oxygen saturation could be due to the generally low blood hematocrit levels in the study patients, as the blood oxygen-carrying capacity increases linearly and the blood viscosity, which affects flow resistance, increases exponentially with increasing blood hematocrit levels, leading to an overall decrease of the blood oxygen-carrying capacity once the hematocrit level exceeds 50% [11, 15, 16]. But in this context, it should be emphasized that other variables such as the caliber of the flap pedicle and the anatomy of the flap vasculature have a greater influence on flap blood and hemoglobin oxygen saturation, as the flap pedicle length and diameter are generally different between flap types and directly determine the blood vessel flow and thus also indirectly the hemoglobin oxygen saturation [6, 12, 23–25]. In general, the associations between blood hemoglobin and hematocrit levels and postoperative hemoglobin concentration in RFFFs have no clinical implications, as the effect size indicates only a marginal impact of both on flap perfusion measurement and the threshold values for both are relative values that refer to previously measured levels [6, 7].

The study has several limitations. Flap perfusion was measured at only a single spot in the center of the flap and at only two timepoints, once intraoperatively and once postoperatively. However, the study used available data and adopted a retrospective approach, so the number of measurement spots and the number of timepoints could not be changed, perfusion measurement in the context of flap monitoring with the O2C analysis system is typically performed at one spot in the center of the flap, which supports orientation for subsequent measurements, and data for variables thought to influence flap perfusion, such as mean arterial blood pressure and catecholamine dose administered, were only available at the two time points included in the study [6, 7, 27]. Besides, the influence of other variables, such as differences in patient anatomy (e.g., vessel diameter and length) cannot be ruled out. A further limitation with regard to the validity of the study results is that the blood parameter levels in the microvasculature are not identical to the levels measured in the blood gas analysis, due to, for example, axial cell migration within the microvasculature, and the composition of the blood plasma also influences blood viscosity and flow resistance [28, 29]. Besides, the blood parameter levels determined via arterial blood gas analysis are usually overestimated as compared to those determined via conventional laboratory methods [30]. Another limitation of the study is that deviations in blood hemoglobin and hematocrit levels from the values measured during the time interval between the arterial blood gas analysis and the measurement of flap perfusion cannot be excluded. However, the measurement of flap perfusion was performed at the end of or after the surgical procedure, so major blood loss is unlikely, and patients who underwent blood transfusions or the substitution of colloidal volume between the arterial blood gas analysis and the measurement of flap perfusion were excluded. With regard to the statistical analysis, a lack of power due to the low number of patients included in the study and the separate analysis for each flap type (which was chosen in view of the differences in flap type anatomy and perfusion measurement parameters) should be kept in mind, which conditions the exploratory nature of the study [6, 7, 23–25]. In general, the study only covered the intraoperative and immediate postoperative period, so future studies over a longer postoperative period may provide a more complete picture of the relationship between flap perfusion and blood hemoglobin and hematocrit levels.

## Conclusions

This study did not observe any association between the O2C analysis system measurement parameters flap blood flow and hemoglobin oxygen saturation and blood hemoglobin and hematocrit levels in the intraoperative and immediate postoperative period. This underlines the validity of the predefined absolute threshold values for flap blood flow and hemoglobin oxygen saturation used for decision-making about flap revision in the context of flap monitoring with the O2C analysis system.

## Data Availability

The datasets used and/or analyzed during the current study are under further analysis and are available from the corresponding author on reasonable request.

## Abbreviations

O2C: Oxygen-2-see
RFFF: Radial free forearm flap
ALTF: Anterolateral thigh flap
FFF: Fibula free flap
AU: Arbitrary unit
